# The effects of hospice care on healthcare expenditure among cancer patients

**DOI:** 10.1186/s12913-023-09578-2

**Published:** 2023-08-07

**Authors:** Hoyol Jhang, Wonjeong Jeong, Hyun-Soo Zhang, Dong-Woo Choi, Hyejung Kang, Sohee Park

**Affiliations:** 1 Department of Health Informatics & Biostatistics, Graduate School of Public Health, Yonsei University, 50-1 Yonsei-ro, Seodaemun-gu, Seoul, Seoul, 03722 Republic of Korea; 2 Cancer Knowledge & Information Center, National Cancer Control Institute, National Cancer Center, Goyang, Republic of Korea; 3 Department of Biomedical Informatics, College of Medicine, Yonsei University, Seoul, Republic of Korea; 4 Cancer Big Data Center, National Cancer Control Institute, National Cancer Center, Goyang, Republic of Korea

**Keywords:** Hospice, Healthcare expenditure, Life-sustaining treatment, Cancer, End-of-life decision

## Abstract

**Purpose:**

It is necessary to estimate the hospice usage and hospice-related cost for entire cancer patients using nationwide cohort data to establish a suitable ethical and cultural infrastructure. This study aims to show the effects of hospital hospice care on healthcare expenditure among South Korean cancer patients.

**Methods:**

This study is a retrospective cohort study using customized health information data provided by the National Health Insurance Service. Individuals who were diagnosed with stomach, colorectal, or lung cancer between 2003 and 2012 were defined as new cancer patients, which included 7,176 subjects. Patients who died under hospital-based hospice care during the follow-up period from January 2016 to December 2018 comprised the treatment group. Healthcare expenditure was the dependent variable. Generalized estimating equations was used.

**Results:**

Among the subjects, 2,219 (30.9%) had used hospice care at an average total cost of 948,771 (± 3,417,384) won. Individuals who had used hospice care had a lower odds ratio (EXP(β)) of healthcare expenditure than those who did not (Total cost: EXP(β) = 0.27, 95% confidence intervals (CI) = 0.25–0.30; Hospitalization cost: EXP(β) = 0.32, 95% CI = 0.29–0.35; Outpatient cost: EXP(β) = 0.02, 95% CI = 0.02–0.02).

**Conclusion:**

Healthcare expenditure was reduced among those cancer patients in South Korea who used hospice care compared with among those who did not. This emphasizes the importance of using hospice care and encourages those hesitant to use hospice care. The results provide useful insights into both official policy and the existing practices of healthcare systems.

**Supplementary Information:**

The online version contains supplementary material available at 10.1186/s12913-023-09578-2.

## Introduction

Cancer is a leading cause of death worldwide and a major driver of the demand for healthcare [1]. In South Korea, cancer is currently one of the foremost public health concerns [2]. The overall cancer incidence rate in South Korea increased by approximately 3.5% per year until 2011; thereafter, the incidence rate declined by 2.7% per year until 2017 [3]. Although these rates have decreased slightly, the burden of cancer continues to grow with the increasing age of the population [2]. The economic burden of cancer among patients aged 60 years and over also continues to gradually grow [4]. As well-dying (dying with dignity) has become an important palliative care aim, palliative and hospice referrals for terminally ill cancer patients are also becoming increasingly significant [5].

Hospice care focuses on the quality of life of people who are experiencing an advanced, life-limiting illness, and that of their caregivers [6]. Hospice care addresses the pain, symptoms, and stress associated with serious illnesses during a patient’s terminal phase, with a life expectancy of about six months or less if the disease runs its natural course [7]. The goal of hospice care is to provide comfort through pain and symptom management, as well as psychosocial and spiritual support when curative treatment modalities are no longer beneficial or effective [7, 8]. However, the rate of hospice use in South Korea is significantly lower than that of the top 10 countries worldwide [9]. Therefore, as Korea has attempted to expand its life-sustaining treatment system, the budget for 2019 increased by 102.6% compared to 2018 [10]. Moreover, the Law on Hospice and Palliative Care and the Determination of Life-Sustaining Treatment for Terminally Ill Patients (Act No. 14,013) (henceforth, Determination of Life-Sustaining Treatment Act) was enacted in January 2016, and the determination of Life-Sustaining Treatment Act came into effect in February 2018 [11, 12]. However, a realistic reimbursement system is required to ensure the financial stability of terminally ill patients [13].

Medical use escalates rapidly among terminally ill cancer patients, leading to increased medical expenditures. As found in a previous study, for the three months before death, medical expenses accounted for 50.4% of the medical expenses for one year before death, and reached their peak one month before death, being nearly twice as much as that in the previous month [14]. However, those who used hospice care experienced an overall reduction in expenditure compared with those who did not; those who had lung cancer or colorectal cancer experienced the greatest reduction in hospital use [15]. The reason for the low healthcare expenditure associated with hospice care is that patients and their families are presented with treatment goals and are required to choose the appropriate treatment to meet their current goals; this reduces the healthcare expenditure on life-sustaining treatment [14]. A decreased rate of hospitalization is considered an indicator of good quality end-of-life care and is highly associated with increased patient satisfaction, which is an important goal of hospice [16].

Therefore, to establish an ethical and cultural infrastructure, it is necessary to estimate the hospital hospice usage and hospice-related cost for entire cancer patient using nationwide cohort data. In this study, we hypothesize that cancer patients who use hospital-based hospice care have lower healthcare expenditures compared with non-hospice cancer patients, that is, patients who do not undergo medical care that prolongs life. Consequently, this study examines the effects of hospice care on healthcare expenditure among South Korean cancer patients.

## Methods

### Data and study participants

This study uses customized health information data provided by the National Health Insurance Service (NHIS). The NHIS collects and manages health information for all registered Koreans and provides the collected data only for research purposes. Health information data include sociodemographic characteristics and information regarding births, deaths, medical use, and examinations. When a researcher selects and applies the desired data type, the NHIS processes and provides the data according to the request [17].

In this study, 2002 was designated as the wash-out period. Individuals who were diagnosed with stomach, colorectal, or lung cancer between 2003 and 2012 using the International Classification of Disease, 10th revision (ICD-10) code of C16 (stomach cancer), C18, C19, C20 (colorectal cancer), and C33, C34 (lung cancer) were defined as new cancer patients. A retrospective cohort was constructed in which a follow-up was performed from the time of diagnosis to the end of the cohort (December 31, 2018) or the time of death. Patients who died under hospital hospice care during the follow-up period from January 2016 to December 2018 comprised the patient group within the cohort. When checking the boxplot for the average daily medical cost after matching for hospice use, a value of 100 million won or more showed a very large extreme value; therefore, extreme values were excluded. Furthermore, subjects who died on the day of their hospice admittance were also excluded. This study adhered to the tenets of the Declaration of Helsinki and was based on the routinely collected administrative and claims data. This study was reviewed and approved by the Institutional Review Board of the Yonsei University Health System (IRB approval number: 4-2021-0374). The need for written informed consent was waived by NHIS ethics committee due to retrospective nature of the study.

### Variables

#### Hospice

In this study, we defined the subjects who received hospital hospice care as those whose behavior code in the NHIS data included “WA,” “WB,” “WC,” “WD,” “WE,” “WF,” “WG,” “WH,” “WJ,” “WK,” “WL,” “WM,” “WN,” and “WO,” between January 2016 to December 2018. The codes “WA,” “WB,” “WC,” “WJ,” “WK,” and “WL” referred to hospital-based hospital care with caregiving, while “WD,” “WE,” “WF,” “WM,” “WN,” and “WO” referred to care without caregiving. Additionally, “WG” referred to comprehensive care, and “WH” referred to end-of life care. Additionally, the control group consisted of subjects who did not receive hospice care and had similar characteristics in the period of 15 days before death as those of subjects who received hospice care.

#### Average daily medical cost

The average daily medical cost of the hospice subjects was defined as the sum of the medical expenses incurred during the period from hospice admittance to the time of death divided by the period. Additionally, the average daily medical expenses of the control group were defined as the sum of the medical expenses incurred during the period from the hospice admittance of the matched hospice subject to the time of death of the control group subject divided by the period. Furthermore, according to the type of treatment, the average daily medical expenses at hospitalization and average outpatient medical expenses were classified. The medical cost of hospice care was expressed in Korean dollars \ (won) ($1 = \1,189.90 on November 24, 2021).

#### Confounding variable

In this study, the control variables included sex, age, the Charlson comorbidity index (CCI), social security, income, region, history of cancer, and the period from diagnosis to death.

Sex was divided into male and female, and age was divided into 50, 50–54, 55–59, 60–64, 65–69, 70–74, 75–79, and ≥ 80 years. According to previous studies, hospice-related studies used subjects aged 50 years or older [18], and cancer patients were divided into age groups of 5 years, so this study was also applied [19]. The CCI is a value obtained by selecting 17 diseases that predict one-year mortality, giving weights of 1, 2, 3, and 6 points according to the relative risk of each disease, and adding them together [20]. In this study, the CCI values were classified as 0, 1, 2, 3, 4, and 5 or higher. Social security was divided into regional insurance, corporate insurance, and medical aid, and income was divided into quintiles (low, lower-middle, middle, upper-middle, and high). Region was divided into capital city (Seoul), metropolitan, city, and rural area, and history of cancer was divided into gastric cancer, colorectal cancer, and lung cancer. The period from diagnosis to death was defined as number of days.

#### Statistical analysis

In this study, 1:N propensity score matching was performed for hospice use (N = 1, 2, 3). The variables used for matching were sex, age, the CCI, social security, income, region, history of cancer, and the period from diagnosis to death. A frequency analysis was then performed using the chi-square and Wilcoxon rank-sum tests for hospice use and the control variables, respectively. The average daily medical cost of the subject was expressed as the mean and standard deviation (SD). Generalized estimating equations (GEE) were used to determine the relationship between hospice use and average daily medical costs, and the results were expressed as expected values and 95% confidence intervals (CI). Additionally, a subgroup analysis according to sex, age, the CCI, social security, income, region, and history of cancer was performed using the GEE. Finally, a frequency analysis was performed on whether hospice care was implemented and the average daily medical cost, according to the last days of life, followed by a subgroup analysis according to the period, performed using the GEE. The significance level for all analyses was 0.05. All data analyses were conducted using SAS Enterprise 7.1 (SAS Institute Inc., Cary, NC, USA).

## Results

As a result of propensity score matching, 1:3 matching included 4,387 people (case: 1,097, control: 3,290, except one extreme value of control), 1:2 matching included 1,635 people (case: 545, control: 1,090), 1:1 matching included 1,154 people (case: 577, control: 577); therefore, 7,176 subjects were selected for the analysis.

Table 1 confirms the general characteristics of the subjects regarding their hospice care use after propensity score matching. A large proportion of the subjects were men (68.1%), and many subjects were in the age group of 65–69 years (66.9%). The CCI scores of 0 and 2 had the highest proportions of 26.9% and 27.4%, respectively. Regarding social security, regional insurance was the most common method (68.3%). Regarding income, the low-income group comprised the largest proportion of the sample (30.5%). A high proportion of the subjects were from cities (41.2%). Colorectal (38.6%) and stomach cancer (38.6%) were the most common cancer histories. The average period from diagnosis to death was 2,800 days among all the subjects, and the mean hospice period was 38 days. Not all variables were statistically significant.

**Table 1:** General characteristics of the study population according to hospice care

Variables		Total		Hospice				P-value
				Yes		No		
		N / Mean	(%) / SD	N / Mean	(%) / SD	N / Mean	(%) / SD	
Total		7,176	(100.0)	2,219	(30.9)	4,957	(69.1)	
**Sex**								0.8451
	Male	4,885	(68.1)	1,507	(30.8)	3,378	(69.2)	
	Female	2,291	(31.9)	712	(31.1)	1,579	(68.9)	
**Age (years**)								0.1410
	< 50	508	(7.1)	163	(32.1)	345	(67.9)	
	50–54	505	(7.0)	167	(33.1)	338	(66.9)	
	55–59	803	(66.9)	276	(28.5)	527	(71.5)	
	60–64	1,090	(33.1)	347	(30.1)	743	(69.9)	
	65–69	1,529	(21.3)	462	(30.2)	1,067	(69.8)	
	70–74	1,472	(20.5)	420	(28.5)	1,052	(71.5)	
	75–79	929	(12.9)	279	(30.0)	650	(70.0)	
	≥ 80	340	(4.7)	105	(30.9)	235	(69.1)	
**Charlson Comorbidity Index**								0.3980
	0	1,930	(26.9)	592	(30.7)	1,338	(69.3)	
	1	1,201	(16.7)	361	(30.1)	840	(69.9)	
	2	1,965	(27.4)	605	(30.8)	1,360	(69.2)	
	3	1,301	(18.1)	393	(30.2)	908	(69.8)	
	4	408	(5.7)	140	(34.3)	268	(65.7)	
	≥ 5	371	(5.2)	128	(34.5)	243	(65.5)	
**Social security**								0.4920
	Insurance (Regional)	4,901	(68.3)	1,530	(31.2)	3,371	(68.8)	
	Insurance (Corporate)	2,222	(31.0)	670	(30.2)	1,552	(69.8)	
	Medical aid	53	(0.7)	19	(35.8)	34	(64.2)	
**Income**								0.5626
	Low	2,192	(30.5)	654	(29.8)	1,538	(70.2)	
	Lower-middle	1,161	(16.2)	371	(32.0)	790	(68.0)	
	Middle	1,327	(18.5)	403	(30.4)	924	(69.6)	
	Upper-middle	1,279	(17.8)	399	(31.2)	880	(68.8)	
	High	1,217	(17.0)	392	(32.2)	825	(67.8)	
**Region**								0.0881
	Capital city	1,498	(20.9)	494	(33.0)	1,004	(67.0)	
	Metropolitan	1,872	(26.1)	591	(31.6)	1,281	(68.4)	
	City	2,955	(41.2)	892	(30.2)	2,063	(69.8)	
	Rural area	851	(11.9)	242	(28.4)	609	(71.6)	
**Cancer**								0.3048
	Stomach	2,767	(38.6)	828	(29.9)	1,939	(70.1)	
	Colorectal	2,771	(38.6)	882	(31.8)	1,889	(68.2)	
	Lung	1,638	(22.8)	509	(31.1)	1,129	(68.9)	
**Period from diagnosis to death** ^**a**^		2,800	956	2,809	992	2,796	939	0.8496
**Hospice period** ^**a**^		38	59	38	59	-	-	-

Note. ^a^ Mean and standard deviation (SD) of the continuous independent variables in this study

Table 2 shows the means and SDs of the subjects’ healthcare expenditure. Regarding the total study population, the average total costs, hospitalization costs, and outpatient costs were 948,771 won (SD = 3,417,384 won), 805,869 won (SD = 2,930,833 won), and 142,902 won (SD = 900,858 won), respectively. Among the hospice subjects, the average total costs, hospitalization costs, and outpatient costs were 309,618 won (SD = 87,034 won), 307,119 won (SD = 89,144 won), and 2,499 won (SD = 9,178 won), respectively. Among the control group, the average total costs, hospitalization costs, and outpatient costs were 1,234,887 won (SD = 4,079,127 won), 1,029,134 won (SD = 3,502,997 won), and 205,753 won (SD = 1,078,003 won), respectively.

**Table 2:** Results of the mean and standard deviation of the study population’s healthcare expenditure

Variables		Healthcare Expenditure												
		Total cost				Hospitalization cost				Outpatient cost				
		Mean	SD			Mean	SD			Mean	SD			
**Total**		948,771	±	3,417,384		805,869	±	2,930,833		142,902	±	900,858		
**Hospice**														
	Yes	309,618	±	87,034		307,119	±	89,144		2,499	±	9,178		
	No	1,234,887	±	4,079,127		1,029,134	±	3,502,997		205,753	±	1,078,003		
**Sex**														
	Male	967,961	±	3,393,461		819,801	±	2,910,975		148,161	±	902,827		
	Female	907,851	±	3,468,234		776,162	±	2,973,155		131,689	±	896,739		
**Age (years**)														
	< 50	1,154,895	±	4,075,737		980,430	±	3,481,041		174,465	±	1,147,916		
	50–54	1,276,575	±	4,454,243		1,011,845	±	3,744,044		264,731	±	1,203,844		
	55–59	1,178,816	±	4,215,924		988,033	±	3,460,121		190,783	±	1,393,098		
	60–64	1,043,629	±	3,776,984		882,271	±	3,214,199		161,357	±	893,228		
	65–69	994,946	±	3,158,468		812,874	±	2,533,814		182,072	±	1,015,836		
	70–74	827,706	±	3,294,221		735,692	±	3,097,017		92,014	±	449,039		
	75–79	603,433	±	1,743,374		548,529	±	1,588,414		54,904	±	311,461		
	≥ 80	566,560	±	1,913,643		539,414	±	1,839,510		27,146	±	148,588		
**Charlson Comorbidity Index**														
	0	984,040	±	3,475,375		821,563	±	2,759,505		162,477	±	1,097,679		
	1	854,181	±	3,245,237		748,074	±	2,986,666		106,107	±	647,347		
	2	1,061,187	±	4,012,289		905,013	±	3,518,916		156,174	±	949,711		
	3	810,709	±	2,176,210		682,835	±	1,808,062		127,874	±	599,227		
	4	878,303	±	3,007,707		714,847	±	2,269,775		163,456	±	1,260,134		
	≥ 5	1,037,725	±	4,137,154		917,742	±	3,862,677		119,984	±	539,831		
**Social security**														
	Insurance (Regional)	996,835	±	3,592,845		845,766	±	3,038,052		151,069	±	973,631		
	Insurance (Corporate)	996,835	±	3,030,485		845,766	±	2,707,323		151,069	±	727,625		
	Medical aid	639,659	±	1,429,064		613,555	±	1,397,822		26,105	±	87,180		
**Income**														
	Low	991,474	±	3,612,863		834,172	±	3,063,353		157,302	±	1,075,116		
	Lower-middle	948,462	±	3,514,503		816,305	±	3,107,973		132,157	±	838,344		
	Middle	818,549	±	2,817,124		703,402	±	2,407,966		115,147	±	717,275		
	Upper-middle	858,767	±	3,049,771		743,059	±	2,779,310		115,708	±	531,769		
	High	1,108,730	±	3,888,717		922,672	±	3,176,295		186,057	±	1,083,148		
**Region**														
	Capital city	890,849	±	2,887,393		763,517	±	2,443,379		127,333	±	745,869		
	Metropolitan	1,151,050	±	4,235,716		981,267	±	3,784,679		169,783	±	879,593		
	City	910,338	±	3,082,234		776,370	±	2,718,565		133,968	±	772,128		
	Rural area	739,214	±	3,353,273		597,016	±	2,159,210		142,198	±	1,446,857		
**Cancer**														
	Stomach	956,324	±	3,578,175		828,404	±	3,132,075		127,921	±	882,543		
	Colorectal	950,959	±	3,392,930		819,051	±	2,897,658		131,908	±	891,417		
	Lung	932,308	±	3,173,896		745,500	±	2,619,367		186,808	±	945,432		

Note. SD, standard deviation

Table 3 confirms the relationship between the participants’ healthcare expenditure and hospice implementation. Compared with the subjects who did not use hospice care, the odds of total medical expenses were 0.27 times lower, hospitalization costs were 0.32 times lower, and outpatient costs were 0.02 times lower among those who used hospice care, which was statistically significant. Additionally, the odds of total medical expenses, inpatient medical expenses, and outpatient medical expenses decreased as age increased, and this was statistically significant in the 70–75, 75–80, and 80 years old or older age groups compared with the under 50 years old age group. Sex, the CCI, social security, income, region, cancer, and the period from diagnosis to death were not statistically significant.

**Table 3:** Results of hospice implementation on the study population’s healthcare expenditure

Variables		Total cost					Hospitalization cost					Outpatient cost				
		EXP(ß)	95% CI				EXP(ß)	95% CI				EXP(ß)	95% CI			
**Hospice**																
	Yes	0.27	(0.25	–	0.30)		0.32	(0.29	–	0.35)		0.02	(0.02	–	0.02)	
	No	1.00					1.00					1.00				
**Sex**																
	Male	1.00					1.00					1.00				
	Female	0.99	(0.90	–	1.09)		1.00	(0.90	–	1.10)		1.01	(0.92	–	1.11)	
**Age (years)**																
	< 50	1.00					1.00					1.00				
	50–54	1.01	(0.80	–	1.28)		0.96	(0.75	–	1.22)		1.20	(0.94	–	1.53)	
	55–59	0.98	(0.79	–	1.21)		0.98	(0.79	–	1.21)		0.89	(0.71	–	1.11)	
	60–64	0.89	(0.72	–	1.09)		0.90	(0.73	–	1.11)		0.79	(0.64	–	0.98)	
	65–69	0.86	(0.70	–	1.04)		0.84	(0.69	–	1.03)		0.88	(0.72	–	1.08)	
	70–74	0.71	(0.58	–	0.87)		0.74	(0.61	–	0.91)		0.55	(0.45	–	0.68)	
	75–79	0.58	(0.47	–	0.71)		0.61	(0.49	–	0.75)		0.44	(0.36	–	0.55)	
	≥ 80	0.54	(0.41	–	0.71)		0.59	(0.45	–	0.78)		0.31	(0.24	–	0.41)	
**Charlson Comorbidity Index**																
	0	1.00					1.00					1.00				
	1	0.94	(0.82	–	1.08)		0.96	(0.83	–	1.10)		0.86	(0.75	–	1.00)	
	2	1.08	(0.96	–	1.22)		1.08	(0.96	–	1.22)		1.10	(0.97	–	1.24)	
	3	0.88	(0.77	–	1.01)		0.87	(0.75	–	0.99)		1.00	(0.87	–	1.14)	
	4	0.93	(0.76	–	1.14)		0.90	(0.73	–	1.10)		1.29	(1.04	–	1.59)	
	≥ 5	1.07	(0.87	–	1.33)		1.12	(0.91	–	1.39)		1.17	(0.94	–	1.44)	
**Social security**																
	Insurance (Regional)	1.00					1.00					1.00				
	Insurance (Corporate)	0.98	(0.89	–	1.09)		0.97	(0.88	–	1.08)		1.02	(0.92	–	1.13)	
	Medical aid	0.78	(0.47	–	1.32)		0.85	(0.50	–	1.43)		0.49	(0.29	–	0.83)	
**Income**																
	Low	1.00					1.00					1.00				
	Lower-middle	0.95	(0.83	–	1.09)		0.98	(0.85	–	1.12)		0.80	(0.69	–	0.92)	
	Middle	0.84	(0.74	–	0.96)		0.86	(0.75	–	0.98)		0.83	(0.72	–	0.95)	
	Upper-middle	0.92	(0.80	–	1.05)		0.94	(0.82	–	1.07)		0.84	(0.73	–	0.96)	
	High	1.17	(1.02	–	1.34)		1.16	(1.01	–	1.33)		1.34	(1.16	–	1.54)	
**Region**																
	Capital city	1.00					1.00					1.00				
	Metropolitan	1.23	(1.08	–	1.41)		1.24	(1.08	–	1.41)		1.23	(1.07	–	1.40)	
	City	1.00	(0.89	–	1.13)		1.00	(0.89	–	1.13)		0.96	(0.85	–	1.09)	
	Rural area	0.90	(0.76	–	1.05)		0.86	(0.73	–	1.01)		1.12	(0.95	–	1.32)	
**Cancer**																
	Stomach	1.00					1.00					1.00				
	Colorectal	1.02	(0.92	–	1.13)		1.01	(0.91	–	1.12)		1.12	(1.01	–	1.24)	
	Lung	0.96	(0.85	–	1.08)		0.90	(0.79	–	1.01)		1.28	(1.13	–	1.46)	
**Period from diagnosis to death**		1.00	(1.00	–	1.00)		1.00	(1.00	–	1.00)		1.00	(1.00	–	1.00)	

Note. CI, confidence interval.

Appendix 1 presents the relationship between the subjects’ medical costs and hospice care, analyzed by subgroup analyses according to sex, age, the CCI, social security, income, region, and history of cancer. Regarding social security, the odds of total medical costs, inpatient medical costs, and outpatient medical costs were significantly lower among subjects who used hospice care compared with among those who did not in all subgroups except the medical aid group.

Appendix 2 confirms the average and SD of the number of subjects and the average daily medical cost according to whether hospice care was performed in the last days of the subject’s life. The total costs of care, inpatient care, and outpatient care were higher on average among participants who did not use hospice care compared with among those who used hospice care.

Table 4 shows the relationship between the subjects’ medical expenses and hospice use based on a subgroup analysis according to the subject’s last days of life. In all periods, the odds of total medical costs, inpatient medical expenses, and outpatient medical expenses were lower and statistically significant among subjects who used hospice care compared with among those who did not.

**Table 4:** Results of a subgroup analysis regarding the relationship between hospice use and healthcare expenditure, according to the last days of life

Variables	Hospice														
	No	Yes													
		Total cost				Hospitalization cost				Outpatient cost					
		**EXP(ß**)	**95% CI**			**EXP(ß**)	**95% CI**			**EXP(ß**)	**95% CI**				
**Period from diagnosis to death**															
Last 30 days of life	1.00	0.19	(0.17	–	0.21)	0.23	(0.20	–	0.25)	0.01	(0.01	–	0.01)		
Last 60 days of life	1.00	0.24	(0.22	–	0.27)	0.28	(0.25	–	0.31)	0.02	(0.01	–	0.02)		
Last 90 days of life	1.00	0.25	(0.23	–	0.28)	0.30	(0.27	–	0.33)	0.02	(0.01	–	0.02)		
Last 6 months of life	1.00	0.27	(0.24	–	0.29)	0.31	(0.28	–	0.35)	0.02	(0.02	–	0.02)		
Last 1 year of life	1.00	0.27	(0.25	–	0.30)	0.32	(0.29	–	0.35)	0.02	(0.02	–	0.02)		

Note. CI, confidence interval

## Discussion

As the rate of hospice care among cancer patients is on the rise, finding how hospice care affects healthcare expenditure is necessary to attempt to delay the process of death and increase the patient’s quality of life [21]. Hospice for cancer patients can lead to significantly improved quality of life, more cost-effective treatment, and prolonged survival time before the end of their lives [22]. Therefore, this study estimates the hospice-related costs using big data for entire nationwide patients with gastric cancer, colorectal cancer, and lung cancer. Our findings show a significant effect of hospice care on reduced healthcare expenditure.

Based on our results, total expenditure, as well as hospitalization and outpatient costs can reduce among those who use hospital-based hospice care compared with among those who do not. While patients aim to minimize their pain and improve their quality of life, the cost aspect of healthcare cannot be ignored. Economic issues are one of the biggest concerns for both patients and their caregivers as the total costs of healthcare continuously increase at the end of a patient’s life. Thus, reducing economic stress can also improve a patient’s quality of life [23]. A previous study has shown that hospice care is more cost-effective than general ward treatment and is considered a cost-effective alternative to such treatment [14].

Our findings suggest that expenditures in the last 30 days of life were lower among those who used hospice care compared with among those who did not. The average monthly medical expenditure (over a year) of 1,243,299 won for non-hospice patients increased to 1,779,527 won in the last 30 days of life. However, this differs for hospice users as the average monthly expenditure increased less significantly from 31,269 won (over a year) to 315,706 won in the last 30 days of life, which is much less than that among those who did not use hospice care. Moreover, a previous study has shown that among those patients who had died, the hospitalization days within one year before their death were most cost-effective when they were treated under hospice palliative care [14, 24].

In South Korea, the proportion of men is about 14% higher than that of women, people aged 60 or older account for 75% of the population, and healthcare expenditure increases with age [25]. Similarly, cancer patients who comply with life-sustaining treatment decisions are younger than 65 years of age, usually live in cities, and have higher incomes than those who do not comply [26]. In this study, men who used hospice care experienced reduced healthcare expenditures compared with men who did not receive hospice care. Moreover, among the subjects in the high-income group, those who used hospice care spent less money than those who did not use hospice care. This shows that using hospice care reduced healthcare expenditure across most socioeconomic statuses.

Efforts are underway to develop and strengthen a hospice care policy to support patients in Korea. Since the introduction of hospital hospice in 2015, home-based hospice and consultative hospice care were separately implemented as program in 2020 and 2022, respectively. This is aimed at expanding the options available to patients with terminally illness, and enabling them to receive hospice services regardless of their location. In terms of the payment system of hospice care, terminally ill cancer patients only have to pay 5% of their total medical expenses after being registered as severe patients under the expanding benefit coverage system for cancer patients [27]. However, due to the low fee schedule, it is difficult to operate hospice palliative care facilities without government support or private donations, and there is a limit to motivating the introduction of new facilities. As fees can reflect the societal values, hospice policy can be an important issue [27].

Our study has several limitations. First, we could not investigate the detailed hospice progress of individual patients as we used claims data. Second, to demonstrate homogeneity, only those who had died were included in the analysis. Third, only patients with stomach, colorectal, or lung cancer were included in this study; however, these are the three most common cancers among both sexes in South Korea [3]. Fourth, due to the limitations of our data, this study only included hospital hospice care and did not cover home hospice care and consultative hospice care were not included. However, it should be noted that these separate programs for home and consultative hospice care were implemented after the analysis period, and therefore the results presented include all individuals who used hospice care within our entire cohort of cancer patients. Lastly, due to a lack of data, potential confounding factors such as smoking status, drinking, and physical activity were not included.

Despite these limitations, our study has several strengths. This study may be meaningful in that it is the first study using entire cancer patients of Korean nationwide retrospective cohort data that investigated hospice care and healthcare expenditure. This study used national cohort data, which could represent almost all South Koreans who had stomach, colorectal, or lung cancer [3]. Furthermore, our findings provide evidence for the use of hospice care and possibly lower the frequency of inappropriate life-sustaining treatment that could burden both patients and their families. Moreover, these results can provide evidence for the development of hospice care policies.

The current study has identified that among entire cancer patients’ data, healthcare expenditure was lower among those who used hospital hospice care compared with among those who did not. Currently, many efforts to revitalize hospice palliative care have been made by increasing the connection rate between hospital and home hospice care. Despite these efforts, the current rate of hospice use is still low. Furthermore, the proportion of cancer deaths in Korea among those using hospice services is reported to be very low at 20.0% as of 2017 [14]; this is low compared with the rate in the United States where 48% of all Medicare decedents in 2016 received one or more days of hospice care and were enrolled in hospice care at the time of their deaths [28]. Considering this, the hospice system in Korea remains underused. Hospice use by cancer patients has advantages such as reduced pain intensity, reduced symptom burden, and reduced psychological pain [29]. Also, like the results of this study, it has a positive advantage in terms of cost. Therefore, it is necessary to expand the use of hospice so that cancer patients can experience various advantages and live a better life.

With the introduction of separate programs for home-based hospice care and consultative hospice care have become separate programs in 2020 and 2022, respectively, it is expected that further research will be conducted. While our study only focused on hospital-based hospice care, it is meaningful in that it estimated hospice-related costs for entire cancer patients. This emphasizes the importance of hospice palliative care, provides support for patients who are hesitant to use hospice care, and offers useful insights into both official policy and the existing practices of healthcare systems.

### Electronic supplementary material

Below is the link to the electronic supplementary material.

Supplementary Material 1

## Data Availability

All datasets were available at the National Health Insurance Service (NHIS) database that is available upon request after review of NHIS processes. The datasets used and/or analyzed during the current study are available from the corresponding author on reasonable request.
