# Supplementary material for: The effects of hospice care on healthcare expenditure among cancer patients
Source: BMC Health Serv Res. 2023 Aug 7;23:831. doi: 10.1186/s12913-023-09578-2 (PMC10405473; doi:10.1186/s12913-023-09578-2)
Supplement: Supplementary file 1 — Supplementary Material 1 [file 12913_2023_9578_MOESM1_ESM.docx]

| **Appendix 1 Subgroup Analysis of the healthcare expenditure, according to hospice ^a^** | | | | | | | | | | | | | | | | | | | |
| --- | --- | --- | --- | --- | --- | --- | --- | --- | --- | --- | --- | --- | --- | --- | --- | --- | --- | --- | --- |
|  | | **Hospice** | | | | | | | | | | | | | | | | | |
|  |  | **No** |  | **Yes** | | | | | | | | | | | | | | | |
|  |  |  |  | **Total Cost** | | | |  | **Hospitalization Cost** | | | |  | **Outpatient Cost** | | | | |  |
|  |  | **EXP(ß)** |  | **EXP(ß)** | **95% CI** | | |  | **EXP(ß)** | **95% CI** | | |  | **EXP(ß)** | **95% CI** | | |  |  |
|  |  |  |  |  | **Lower** |  | **Upper** |  |  | **Lower** |  | **Upper** |  |  | **Lower** |  | **Upper** |  |  |
| **Sex** | |  |  |  |  |  |  |  |  |  |  |  |  |  |  |  |  |  |  |
|  | Male | 1.00 |  | 0.27 | (0.24 | - | 0.30) |  | 0.31 | (0.28 | - | 0.35) |  | 0.02 | (0.02 | - | 0.02) |  |  |
|  | Female | 1.00 |  | 0.31 | (0.26 | - | 0.36) |  | 0.36 | (0.30 | - | 0.42) |  | 0.03 | (0.02 | - | 0.03) |  |  |
| **Age** | |  |  |  |  |  |  |  |  |  |  |  |  |  |  |  |  |  |  |
|  | <50 | 1.00 |  | 0.34 | (0.23 | - | 0.50) |  | 0.40 | (0.27 | - | 0.59) |  | 0.02 | (0.01 | - | 0.03) |  |  |
|  | 50-54 | 1.00 |  | 0.25 | (0.17 | - | 0.36) |  | 0.31 | (0.21 | - | 0.45) |  | 0.01 | (0.01 | - | 0.02) |  |  |
|  | 55-59 | 1.00 |  | 0.21 | (0.16 | - | 0.28) |  | 0.26 | (0.19 | - | 0.34) |  | 0.01 | (0.01 | - | 0.02) |  |  |
|  | 60-64 | 1.00 |  | 0.25 | (0.20 | - | 0.32) |  | 0.30 | (0.23 | - | 0.38) |  | 0.02 | (0.01 | - | 0.02) |  |  |
|  | 65-69 | 1.00 |  | 0.27 | (0.22 | - | 0.33) |  | 0.34 | (0.27 | - | 0.42) |  | 0.01 | (0.01 | - | 0.01) |  |  |
|  | 70-74 | 1.00 |  | 0.37 | (0.30 | - | 0.47) |  | 0.43 | (0.34 | - | 0.54) |  | 0.03 | (0.02 | - | 0.04) |  |  |
|  | 75-79 | 1.00 |  | 0.52 | (0.40 | - | 0.68) |  | 0.57 | (0.43 | - | 0.75) |  | 0.08 | (0.06 | - | 0.10) |  |  |
|  | ≥ 80 | 1.00 |  | 0.57 | (0.36 | - | 0.92) |  | 0.61 | (0.37 | - | 0.99) |  | 0.11 | (0.07 | - | 0.16) |  |  |
| **Charlson Comorbidity Index (CCI)** | |  |  |  |  |  |  |  |  |  |  |  |  |  |  |  |  |  |  |
|  | 0 | 1.00 |  | 0.29 | (0.24 | - | 0.35) |  | 0.34 | (0.28 | - | 0.41) |  | 0.02 | (0.02 | - | 0.03) |  |  |
|  | 1 | 1.00 |  | 0.31 | (0.24 | - | 0.39) |  | 0.36 | (0.28 | - | 0.45) |  | 0.02 | (0.02 | - | 0.03) |  |  |
|  | 2 | 1.00 |  | 0.26 | (0.22 | - | 0.31) |  | 0.31 | (0.26 | - | 0.38) |  | 0.02 | (0.01 | - | 0.02) |  |  |
|  | 3 | 1.00 |  | 0.33 | (0.26 | - | 0.41) |  | 0.40 | (0.32 | - | 0.50) |  | 0.02 | (0.02 | - | 0.03) |  |  |
|  | 4 | 1.00 |  | 0.55 | (0.36 | - | 0.82) |  | 0.63 | (0.42 | - | 0.95) |  | 0.06 | (0.04 | - | 0.10) |  |  |
|  | ≥5 | 1.00 |  | 0.34 | (0.22 | - | 0.53) |  | 0.38 | (0.25 | - | 0.60) |  | 0.05 | (0.03 | - | 0.08) |  |  |
| **Social security** | |  |  |  |  |  |  |  |  |  |  |  |  |  |  |  |  |  |  |
|  | Insurance (Regional) | 1.00 |  | 0.26 | (0.23 | - | 0.29) |  | 0.31 | (0.27 | - | 0.34) |  | 0.02 | (0.02 | - | 0.02) |  |  |
|  | Insurance (Corporate) | 1.00 |  | 0.32 | (0.27 | - | 0.38) |  | 0.38 | (0.32 | - | 0.45) |  | 0.03 | (0.02 | - | 0.03) |  |  |
|  | Medical aid | 1.00 |  | 1.22 | (0.26 | - | 5.81) |  | 1.31 | (0.27 | - | 6.30) |  | 0.26 | (0.09 | - | 0.75) |  |  |
| **Income** | |  |  |  |  |  |  |  |  |  |  |  |  |  |  |  |  |  |  |
|  | Low | 1.00 |  | 0.28 | (0.23 | - | 0.33) |  | 0.33 | (0.28 | - | 0.40) |  | 0.02 | (0.01 | - | 0.02) |  |  |
|  | Lower middle | 1.00 |  | 0.31 | (0.24 | - | 0.39) |  | 0.36 | (0.29 | - | 0.47) |  | 0.02 | (0.02 | - | 0.03) |  |  |
|  | Middle | 1.00 |  | 0.35 | (0.28 | - | 0.44) |  | 0.40 | (0.32 | - | 0.51) |  | 0.03 | (0.02 | - | 0.04) |  |  |
|  | Upper middle | 1.00 |  | 0.34 | (0.27 |  | 0.42) |  | 0.40 | (0.31 |  | 0.50) |  | 0.02 | (0.02 |  | 0.03) |  |  |
|  | High | 1.00 |  | 0.24 | (0.19 | - | 0.30) |  | 0.28 | (0.22 | - | 0.36) |  | 0.02 | (0.02 | - | 0.03) |  |  |
| **Region** | |  |  |  |  |  |  |  |  |  |  |  |  |  |  |  |  |  |  |
|  | Capital City | 1.00 |  | 0.30 | (0.24 | - | 0.37) |  | 0.35 | (0.28 | - | 0.43) |  | 0.03 | (0.02 | - | 0.03) |  |  |
|  | Metropolitan | 1.00 |  | 0.25 | (0.20 | - | 0.30) |  | 0.29 | (0.24 | - | 0.35) |  | 0.02 | (0.01 | - | 0.02) |  |  |
|  | City | 1.00 |  | 0.29 | (0.25 | - | 0.34) |  | 0.34 | (0.30 | - | 0.40) |  | 0.02 | (0.02 | - | 0.02) |  |  |
|  | Rural | 1.00 |  | 0.44 | (0.33 | - | 0.58) |  | 0.52 | (0.39 |  | 0.70) |  | 0.04 | (0.03 |  | 0.05) |  |  |
| **Cancer** | |  |  |  |  |  |  |  |  |  |  |  |  |  |  |  |  |  |  |
|  | Stomach | 1.00 |  | 0.29 | (0.25 | - | 0.34) |  | 0.34 | (0.29 | - | 0.39) |  | 0.03 | (0.02 | - | 0.03) |  |  |
|  | Colorectal | 1.00 |  | 0.27 | (0.23 | - | 0.32) |  | 0.32 | (0.27 | - | 0.37) |  | 0.02 | (0.02 | - | 0.03) |  |  |
|  | Lung | 1.00 |  | 0.32 | (0.26 | - | 0.39) |  | 0.40 | (0.32 | - | 0.48) |  | 0.02 | (0.01 | - | 0.02) |  |  |
| **^a^ The reference of each cost (Total cost, Hospitalization cost, Outpatient cost) were no-hospice group (EXP(ß)=1.00) each** | | | | | | | | | | | | | | | | | | | |

| **Appendix 2. Results of Mean and SD of the study populations' healthcare expenditure according to the last days of life** | | | | | | | | | | | | | | | | | | | | | | | | | |
| --- | --- | --- | --- | --- | --- | --- | --- | --- | --- | --- | --- | --- | --- | --- | --- | --- | --- | --- | --- | --- | --- | --- | --- | --- | --- |
| **Variables** | **Hospice** | | **Non-hospice** | |  | **Total** | | | | | |  | **Hospitalization Cost** | | | | | |  | **Outpatient Cost** | | | | | |
|  |  |  |  |  |  | **Hospice** | | | **Non-hospice** | | |  | **Hospice** | | | **Non-hospice** | | |  | **Hospice** | | | **Non-hospice** | | |
|  | **N** | **(%)** | **N** | **(%)** |  | **Mean** |  | **SD** | **Mean** |  | **SD** |  | **Mean** |  | **SD** | **Mean** |  | **SD** |  | **Mean** |  | **SD** | **Mean** |  | **SD** |
| **Period from diagnosis to death** | | | | | | | | | | | | | | | | | | | | | | | | | |
| Last 30 days of life | 1,442 | (31.2) | 3,186 | (68.8) |  | 315,706 | ± | 75,008 | 1,779,527 | ± | 4,983,524 |  | 313,809 | ± | 75,844 | 1,480,145 | ± | 4,283,393 |  | 2,765 | ± | 8,993 | 299,933 | ± | 1,331,020 |
| Last 60 days of life | 1,887 | (31.1) | 4,187 | (68.9) |  | 316,485 | ± | 78,402 | 1,433,480 | ± | 4,406,558 |  | 314,449 | ± | 79,689 | 1,193,583 | ± | 3,785,576 |  | 2,862 | ± | 8,581 | 240,451 | ± | 1,169,363 |
| Last 90 days of life | 2,027 | (31.0) | 4,512 | (69.0) |  | 315,920 | ± | 80,910 | 1,345,260 | ± | 4,258,390 |  | 313,749 | ± | 82,503 | 1,120,811 | ± | 3,657,654 |  | 2,972 | ± | 8,633 | 225,000 | ± | 1,128,015 |
| Last 6 months of life | 2,152 | (31.0) | 4,801 | (69.0) |  | 313,493 | ± | 83,132 | 1,272,635 | ± | 4,139,322 |  | 311,167 | ± | 84,997 | 1,060,593 | ± | 3,554,942 |  | 3,099 | ± | 8,772 | 212,591 | ± | 1,094,693 |
| Last 1 year of life | 2,204 | (30.9) | 4,924 | (69.1) |  | 310,269 | ± | 86,366 | 1,243,299 | ± | 4,091,473 |  | 307,837 | ± | 88,326 | 1,036,253 | ± | 3,513,631 |  | 3,188 | ± | 8,832 | 207,591 | ± | 1,081,390 |
